# Growth factor concentrations and their placental mRNA expression are modulated in gestational diabetes mellitus: possible interactions with macrosomia

**DOI:** 10.1186/1471-2393-10-7

**Published:** 2010-02-09

**Authors:** Oussama Grissa, Akadiri Yessoufou, Inès Mrisak, Aziz Hichami, Daniel Amoussou-Guenou, Abir Grissa, François Djrolo, Kabir Moutairou, Abdelhedi Miled, Hédi Khairi, Monia Zaouali, Iheb Bougmiza, Aabdelkarim Zbidi, Zouheir Tabka, Naim A Khan

**Affiliations:** 1 University of Burgundy, UPRES EA4183 Lipids and Cell Signaling, Faculty of Life Sciences, Dijon, France; 2 Department of Physiology and Functional Exploration, University Hospital Farhat Hached 4000 Sousse, Tunisia; 3 Laboratory of Cell Biology and Physiology (ISBA/FAST), University of Abomey-Calavi, Bénin; 4 Médecine Interne, Service d'Endocrinologie, Centre National Hospitalier et Universitaire (CNHU) de Cotonou, Faculté des Sciences de la Santé, University of Abomey-Calavi, 01 BP 526 Cotonou, Bénin; 5 Department of Biochemistry, University Hospital Farhat Hached 4000 Sousse, Tunisia; 6 Department of Gynaecology, University Hospital Farhat Hached 4000 Sousse, Tunisia; 7 Department of Community Medicine, University Hospital Farhat Hached 4000 Sousse, Tunisia

## Abstract

**Background:**

Gestational diabetes mellitus (GDM) is a form of diabetes that occurs during pregnancy. GDM is a well known risk factor for foetal overgrowth, termed macrosomia which is influenced by maternal hypergycemia and endocrine status through placental circulation. The study was undertaken to investigate the implication of growth factors and their receptors in GDM and macrosomia, and to discuss the role of the materno-foeto-placental axis in the *in-utero *regulation of foetal growth.

**Methods:**

30 women with GDM and their 30 macrosomic babies (4.75 ± 0.15 kg), and 30 healthy age-matched pregnant women and their 30 newborns (3.50 ± 0.10 kg) were recruited in the present study. Serum concentrations of GH and growth factors, *i.e*., IGF-I, IGF-BP3, FGF-2, EGF and PDGF-B were determined by ELISA. The expression of mRNA encoding for GH, IGF-I, IGF-BP3, FGF-2, PDGF-B and EGF, and their receptors, *i.e*., GHR, IGF-IR, FGF-2R, EGFR and PDGFR-β were quantified by using RT-qPCR.

**Results:**

The serum concentrations of IGF-I, IGF-BP3, EGF, FGF-2 and PDGF-B were higher in GDM women and their macrosomic babies as compared to their respective controls. The placental mRNA expression of the growth factors was either upregulated (FGF-2 or PDGF-B) or remained unaltered (IGF-I and EGF) in the placenta of GDM women. The mRNA expression of three growth factor receptors, *i.e*., IGF-IR, EGFR and PDGFR-β, was upregulated in the placenta of GDM women. Interestingly, serum concentrations of GH were downregulated in the GDM women and their macrosomic offspring. Besides, the expression of mRNAs encoding for GHR was higher, but that encoding for GH was lower, in the placenta of GDM women than control women.

**Conclusions:**

Our results demonstrate that growth factors might be implicated in GDM and, in part, in the pathology of macrosomia via materno-foeto-placental axis.

## Background

Excessive birth weight or foetal macrosomia is a common complication of gestational diabetes mellitus (GDM) and is associated with adverse maternal and infantile outcomes including higher rates of postpartum haemorrhage in mothers, perineal lacerations and increased risk for cesarean delivery [1]. Macrosomia has been defined as a birth weight above either 4 kg or 95th percentile for the gestational age. These infants are at greater risk for foetal asphyxia, shoulder dystocia, birth trauma and neonatal hypoglycemia. Furthermore, macrosomic babies may have an increased susceptibility to obesity and diabetes and/or cardiovascular diseases in the later stage of life [2].

Foetal growth is governed by interactions of genetic, nutritional, hormonal and environmental factors [3]. We have previously shown that the metabolism of lipids/lipoproteins [4,5] and antioxidant status [6,7] are altered in macrosomic newborns and their GDM mothers. We have shown the malfunctioning of T-cells [8,9] and high secretion of adipokines in GDM and their macrosomic infants [10]. Hence, we have hypothesized that the accelerated foetal growth, seen in the infants of GDM mothers, may be due to *in utero *programming, caused by a perturbation in the materno-foeto-placental growth axis [9]. Indeed, the *in utero *insulin concentrations have been shown to influence the induction and activity of various hepatic enzymes associated with fat and carbohydrate metabolism [11]. The role of growth factors has also been suggested in the macrosomia [12]. Roth et al. [13] have documented increased levels of IGF-1 in the cord blood of macrosomic infants born to GDM mothers. Lauszus et al. [14] studied diabetic pregnancy and noted that both IGF-1 and IGF-2 levels were correlated with high birth weight. It is noteworthy that the placenta is an important endocrine organ as, during human pregnancy, it produces numerous hormones which may promote early embryonic growth, [15] and influences the fetus by stimulating the production of IGF-I and insulin [16].

Keeping in view the role of insulin and growth factors in the progression of GDM and macrosomia [17], we further studied, in the present report, the materno-foeto-placental axis by determining the concentrations of several growth factors both in GDM mothers and their macrosomic newborns, and by assessing the expression of mRNA encoding for growth factors (GH, IGF-I, FGF-2, PDGF-B and EGF) and their receptors in the placentas of normal and GDM mothers.

## Methods

### Patients

The subjects were recruited in the Gynaecology Department, Hôpital Universitaire Farhat Hached, Tunisia, between February and August 2007. The study protocol was approved by the Sousse Farhat Hached Hospital Committee for Research on Human Subjects (Tunisia). Informed written consent was obtained from all the subjects/mothers. The pregnant women were 19 to 42 years old. Placentas and cord blood were collected in 60 deliveries divided into 30 GDM pregnancies, which had 30 macrosomic babies, and 30 control non-diabetic mothers, which had 30 normal newborns. Spontaneously vaginally delivered newborns, were immediately weighed after delivery. Babies from GDM mothers, whose birth weight was 2 S.D. greater than the mean birth weight of the control infants, were considered as macrosomic infants. Selected control women had no significant history of illness, no pregnancy-related complications and no risk factor for gestational diabetes including normal glucose tolerance tests during the first and third trimesters of pregnancy. GDM was diagnosis when fasting glucose ≥ 5.5 mmol/l. The severity of GDM was categorized according to the fasting plasma glucose level on the 3-hour 100-gr oral glucose tolerance test (OGTT).

### Anthropometric parameters

Birth weight (BW) and length (BL) were obtained from each neonate immediately after birth. BL and head circumferences were measured with plastic-covered fabric measuring tapes and read to the nearest mm. Based on birth length, the ponderal index was calculated as: birth weight (g)/birth length^3 ^(cm) × 100. The BMI was calculated as birth weight (Kg)/birth length^2 ^(m). The biochemical characteristics of mothers and newborns are shown in Table 1.

**Table 1:** Biochemical characteristics of mothers and their offspring

	Newborns		Mothers	
	Control	Macrosomic	Control	Diabetic
Insulinemia (μUI/ml)	5.43 ± .90	7.50 ± 3.25*	4.99 ± 1.20	10.55 ± 4.80**
HbA1c (%)	-	-	4.0 ± 0.50	6.9 ± 0.45**
Fasting glucose (mmol/l)	5.89 ± 0.80	4.96 ± 0.40	4.57 ± 0.77	6.80 ± 0.66**
Apolipoprotein A1	1.21 ± 0.06	1.17 ± 0.1	2.01 ± 0.29	1.91 ± 0.26
Apolipoprotein B	0.56 ± 0.08	0,4 ± 0,05	1.31 ± 0.25	1.23 ± 0.09
CRP	1.7 ± 033	1.74 ± 0.63	4.60 ± 0.8	5.75 ± 1.42
Total cholesterol (mmol/l)	1.85 ± 0.18	1.92 ± 0,12	5.74 ± 0,21	5.19 ± 0.32
HDL-cholesterol (mmol/l)	0.83 ± 0.06	0.92 ± 0.06	2.25 ± 0.16	2.20 ± 0.12
LDL-cholesterol (mmol/l)	0.74 ± 0.15	0.64 ± 0.05	2.61 ± 0.26	2.16 ± 0.26
Triglycerides (mmol/l)	0.57 ± 0.05	0.50 ± 0.01	1.92 ± 0.14	2.48 ± 0.15*
Uric acid	283.86 ± 16.85	305.93 ± 19.29	257.92 ± 15.66	326.45 ± 29.39*
Proteins	49 ± 1.40	51.38 ± 1.67	58.60 ± 0.95	55.50 ± 1.44**
BMI (Kg/m^2^)	13.44 ± 0.30	35.80 ± 0.70*	23.15 ± 2.30	24.90 ± 2.90
Aspartate aminotransferase (UI/l)	36.76 ± 3.27	41.33 ± 6.04	30.02 ± 4.19	21.23 ± 2.57
Alanine aminotransferase (UI/l)	12.67 ± 1.49	11.85 ± 2.10	16.45 ± 2.5	8.25 ± 1.49**
Mode of delivery	-	-	Spontaneous	Spontaneous

Values are means ± SD. n = 60 control mothers and babies; n = 60 gestational diabetic mothers and macrosomic babies. Significant difference between diabetic mothers or macrosomic newborns and their corresponding controls is as follow: *p < 0.05, **p < 0.001.

### Blood sample collection

From each patient or control subject, maternal blood was collected from arm vein after delivery of the baby but before placenta delivery. Cord blood samples were obtained from the umbilical vein immediately after delivery. Fasting venous blood samples were collected in tubes containing or not EDTA to obtain plasma and serum, respectively. Serum or plasma was obtained by centrifugation (1000 gx20 min). Plasma was immediately used for glucose and HbA1c determinations. Serum was aliquoted and frozen at -80°C for further determinations of insulin, GH, IGF-I, IGF-BP3, FGF-2, EGF and PDGF-B concentrations by ELISA (Peprotech Paris, France). Lipid levels were determined by using enzymatic methods, according to the instructions furnished with the kit (Boehringer, Mannheim, Germany).

### Determination of insulin, GH and growth factor concentrations

Serum concentrations of insulin, GH, IGF-I and IGF-BP3 were estimated using non-extraction IRMA Kit (DSL Texas, USA). Serum concentrations of FGF-2, EGF and PDGF-B were estimated using ELISA Immunotech Kit (Peprotech Paris, France).

### Determination of plasma glucose, HbA1c and apolipoprotein levels

Serum triglycerides, total cholesterol and uric acid levels were determined by using enzymatic methods. Plasma fasting glucose was determined by glucose oxidase method using glucose analyzer (Beckman Instruments, Fullerton, CA, USA). Apolipoproteins A1 and B were determined by using spectrophotometer.

### Detection of mRNA of GH, growth factors and their receptors by quantitative RT-PCR

Using RT-qPCR, we evaluated the expression of mRNA of growth hormone (GH), growth factors and their receptors, *e.g*., insulin-like growth factor-I (IGF-I); IGF-I receptor (IGF-IR); fibroblast growth factor-2 (FGF-2); FGF-2 receptor (FGF-2R); platelet-derived growth factor-B (PDGF-B); PDGF receptor-β (PDGFR-β); epidermal growth factor (EGF) and EGF receptor (EGFR) and IGF binding protein-3 (IGF-BP3).

Only one placental tissue from each subject was taken, washed and rinsed in sterile PBS, and immediately plunged into liquid nitrogen and stored at -80°c until the extraction of total RNA. Total RNA was extracted from placental tissue by using Trizol. One μg of total RNA was reverse transcribed with Super script II RNAse H-reverse transcriptase. Real-time PCR was performed on the iCycler iQ real time detection system and amplification was undertaken by using SYBER Green I detection. Oligonucleotide primers, used for mRNA analysis, were based on the sequences of human genes in Gene bank database. The sequences of the PCR primers used are as follows: FGF-2, forward, 5'-CATACAGCAGCAGCCTAGCAAC-3' and reverse, 5'-TTCGGCAACAGCACACAAATCC-3'; EGF, forward, 5'-TCTGCGTGGTGGTGCTTGTC-3' and reverse, 5'-CCTGCGACTCCTCACATC TCTG-3'; PDGF-B, forward, 5'-CAAGACGGCACTGAAGGAGACC-3' and reverse, 5'-GAGACA GACGGACGAGGGAAAC-3'; EGFR, forward, 5'-GAGGGTGAGCCAAGGGAGTTTG-3' and reverse, 5'-GGCAGGTCTTGACGCAGTGG-3'; IGF-1, forward, 5'-CACCATGTCCTCCTCGCAT CTC-3' and reverse, 5'-CCGACTGCTGGAGCCATACC-3'; IGF-BP3, forward, 5'-GGTCCCTGCC GTAGAGAAATGG-3' and reverse, 5'-CCCCGCTTCCTGCCTTTGG-3'; PDGFβ-R, forward, 5'-C GCAGCAGTGAGAAGCAAGC-3' and reverse, 5'-TAGTCCACCAGGTCTCCGTAGC-3'; IGF-1R, forward, 5'-GCCTTGGTCTCCTTGTCCTTCC-3' and reverse, 5'-GTTGCGGTGGTCCCAGTCC-3'; GH, forward, 5'-CCGACACCCTCCAACAGGGA-3' and reverse, 5'-CCTTGTCCATGTCCTTCCTG-3'; GHR, forward, 5'-GGTGAAGGATGGCGACTCTGG-3' and reverse, 5'-TGGATAACACTGGGC TGCTGAG-3'; FGF-2R, forward, 5'-CCCACCGCAGGCTGAAGG-3' and reverse, 5'-CACGACCA GGCAGATGAAACG-3'. Relative quantification of mRNA in different groups was determined as follows: ΔΔCt = ΔCt of gene of interest - ΔCt of 18 S. ΔCt = Ct of Macrosomic - Ct of control. Relative quantity (RQ) was calculated as follows: RQ = (1+E) (-ΔΔCt).

### Statistical analysis

All results are expressed as mean ± standard deviation (SD). Statistical significance of the differences between groups was performed by one-way ANOVA, followed by LSD test. Differences with p < 0.05 were considered to be significant. Simple correlations were assessed by Spearman's rank test.

Multiple regression analysis was carried out by using SPSS (version 15). The dependent variable (BW = birth weight) was normally distributed. Pearson correlation coefficients (r) were determined by the associations between BW and growth factors [PDGF, IGF-BP3, FGF-2, EGF, GH and IGF-1]. A linear regression model was used to evaluate the independent variables explaining the variations in BW. Candidate variables were stepped into the model with a stepwise selection method. To determine entry and removal from the model, significant levels of 0.15 and 0.05 were used, respectively. BW reference equation was evaluated in two groups of 60 gestational mothers, diabetics or not and their new born babies, macrosomic or not.

## Results

### Blood HbA1c, insulin and glucose levels

Plasma HbA1c levels were statistically higher in GDM women than control mothers (Table 1). GDM exhibited higher fasting glycemia and insulinemia compared with healthy pregnant mothers. The macrosomic babies, as well as their age matched controls, were normoglycemic, but the formers were hyperinsulinemic (Table 1).

### Serum biochemical parameters

Triglyceride (TG) levels were higher in GDM mothers compared to the control women. HDL-, LDL- and total-cholesterol were not altered in macrosomic infants and their mothers compared to respective control subjects (Table 1). Serum protein and alanine aminotransferase levels were decreased whereas uric acid concentrations increased in GDM mothers. CRP and apolipiprotein A1 and B remained unchanged in GDM women and macrocosmic babies compared to their respective controls. Serum protein and alanine aminotransferase levels were decreased whereas uric acid concentrations increased in GDM mothers. In fact, a concomitant increase in uric acid, alanine aminotransferase and protein concentrations indicates a situation of pre-eclampsia. Since protein concentrations were lower in GDM subjects, it indicated that there was no renal complication in these subjects.

### Anthropometric Data

There were 14 males and 16 females among control neonates and 18 males and 12 females among macrosomic babies. BW, BL, HC and CP were, respectively, 3.50 ± 0.10 Kg, 49.00 ± 0.32 cm, 13.44 ± 0.30 (Kg/m^2^), and 34.17 ± 0.21 cm in control neonates; and 4.75 ± 0.15 Kg, 52.22 ± 0.31 cm, 35.80 ± 0.70 (Kg/m^2^) and 35.87 ± 0.29 cm in macrosomic babies. Macrosomic neonates had significantly greater BW and BL than control neonates.

### Serum growth factor concentrations and expression of mRNA of growth factor receptors

**Figure 1:**
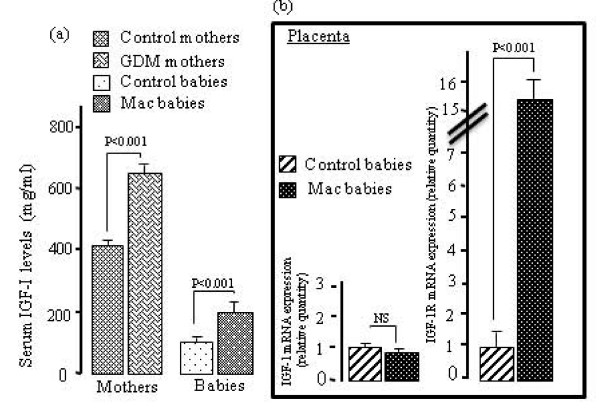
**(a) Serum IGF-I concentrations, and (b) expression of placental mRNA of IGF-I and IGF-IR in gestational diabetic mothers and their babies**. Serum IGF-I concentrations and mRNA expression by RT-PCR were assessed as described in the section of the methods. Values are means ± SD. NS = insignificant differences. n = 60 control mothers and babies; n = 60 gestational diabetic mothers and macrosomic babies.

**Figure 2:**
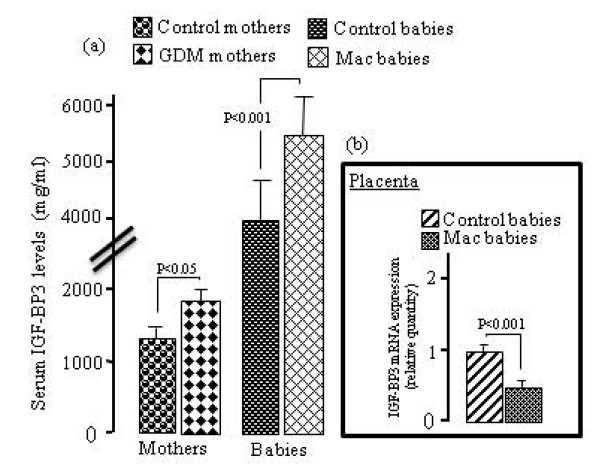
**(a) Serum IGF-BP3 concentrations and (b) expression of placental IGF-BP3 mRNA in gestational diabetic mothers and their babies**. Serum IGF-BP3 concentrations and mRNA expression by RT-PCR were assessed as described in the section of the methods. Values are means ± SD. n = 60 control mothers and babies; n = 60 gestational diabetic mothers and macrosomic babies.

**Figure 3:**
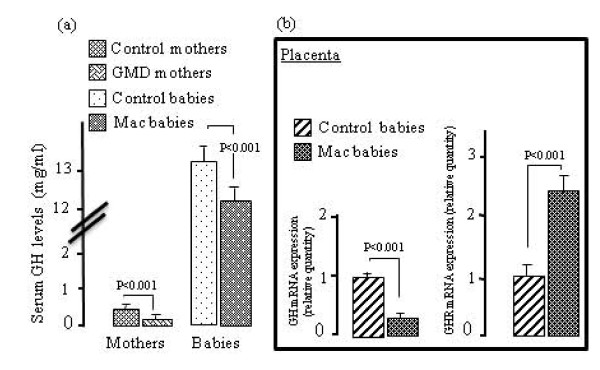
**(a) Serum GH concentrations, and (b) expression of placental mRNA of GH and GHR in gestational diabetic mothers and their babies**. Serum GH concentrations and mRNA expression by RT-PCR were assessed as described in the section of the methods. Values are means ± SD. n = 60 control mothers and babies; n = 60 gestational diabetic mothers and macrosomic babies.

**Figure 4:**
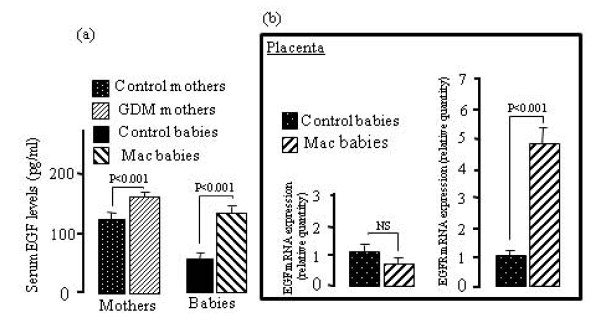
**(a) Serum EGF concentrations, and (b) expression of placental mRNA of EGF and EGFR in gestational diabetic mothers and their babies**. Serum EGF concentrations and mRNA expression by RT-PCR were assessed as described in the section of the methods. Values are means ± SD. NS = insignificant differences. n = 60 control mothers and babies; n = 60 gestational diabetic mothers and macrosomic babies.

**Figure 5:**
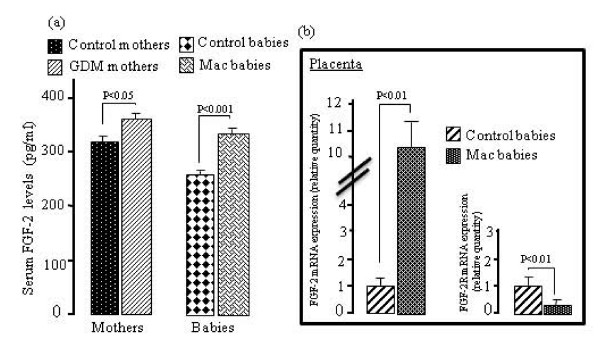
**(a) Serum FGF-2 concentrations, and (b) expression of placental mRNA of FGF-2 and FGF-2R in gestational diabetic mothers and their babies**. Serum FGF-2 concentrations and mRNA expression by RT-PCR were assessed as described in the section of the methods. Values are means ± SD. n = 60 control mothers and babies; n = 60 gestational diabetic mothers and macrosomic babies.

**Figure 6:**
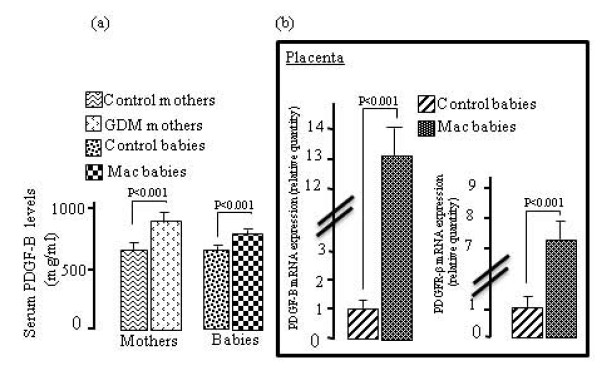
**(a) Serum PDGF-B concentrations, and (b) expression of placental mRNA of PDGF-B and PDGFR-β in gestational diabetic mothers and their babies**. Serum PDGF-B concentrations and mRNA expression by RT-PCR were assessed as described in the section of the methods. Values are means ± SD. n = 60 control mothers/babies; n = 60 gestational diabetic mothers/macrosomic babies.

As compared with non-diabetic mothers and their children, GDM women and their macrosomic newborns exhibited higher serum IGF-I levels. However, no difference was observed in placental IGF-I mRNA expression in GDM, although a significant increases was noticed in placental IGF-IR mRNA expression in these women (Fig. 1). IGF-BP3 concentrations were upregulated in the serum of GDM women and their infants though IGF-BP3 mRNA expression was down-regulated in the placenta of GDM mothers (Fig. 2). The pituitary GH levels in GDM mothers and their macrosomic infants were downregulated. Interestingly, the expression of mRNA encoding for the pituitary GH was downregulated and that for GHR was upregulated in the placenta of GDM women (Fig. 3). Serum EGF levels both in GDM mothers and their macrosomic infants were upregulated though placental EGF mRNA expression remained unaltered in the GDM (Fig. 4). Placental EGFR mRNA expression was higher in GDM mothers than the control women (Fig. 4). Serum FGF-2 levels both in GDM and their macrosomic infants were upregulated as compared to respective controls. FGF-2 mRNA expression was upregulated whereas that of FGF-2R was downregulated in the placenta of GDM (Fig. 5). PDGF-B concentrations and the expression of mRNA for PDGF-B and its receptor, PDGFR-β, were higher in GDM and their macrosomic babies compared to respective controls (Fig. 6).

In this study, we have proposed a model for multiple regression model (Table 2) for the birth weight (BW) and different growth factors. Hence, Table 3 shows the correlation analysis.

**Table 2:** Correlation between birth weight & growth factors

Correlation: birth weight & growth factor	n	r	P
PDGF-NB	60	0.476	0.025*
PDGF-M	60	0.486	0.021*
IGF-BP3-NB	60	0.636	0.01*
IGF-BP3-M	60	0.150	0.502
FGF-2-NB	60	0.626	0.01*
FGF-2-M	60	0.181	0.418
GH-NB	60	0.078	0.724
GH-M	60	0.310	0.159
EGF-NB	60	0.626	0.001**
EGF-M	60	0.181	0.418
IGF-1-NB	60	0.31	0.159
IGF-1-M	60	0.253	0.255

*p < 0.05, **p < 0.001, for abbreviations, see the Table 3.

**Table 3:** Independent variables included in the forward linear stepwise multiple regression model for the birth weight (BW)

VARIABLE	INITIAL MODEL				FINAL MODEL			
	
		95% confidence interval				95% confidence interval		
	
	Mean difference in birth weight (g)	Lower bound	Upper bound	P	Mean difference in birth weight (g)	Lower bound	Upper bound	P
Constant	- 1563	- 7199	4073	0.287	- 1264	- 3124	596	0.170
PDGF-NB	0.659	-3	5	0.594	-	-	-	-
PDGF-M	5.023	2	8	0.002	5.041	3	7	0
IGF-BP3-NB	-0.571	- 2	0	0.177	-	-	-	-
IGF-BP3-M	0.058	0	0	0.277	-	-	-	-
FGF-2-NB	-	-	-	0.074	7.673	5	10	0
FGF-2-M	3.321	- 1	8	0.029	2.743	0	6	0.057
GH-NB	25.320	- 24	75	0.161	-	-	-	-
GH-M	602	- 742	1945	0.100	-	-	-	-
EGF-NB	6.228	1	11	0.001	-	-	-	-
IGF-1-NB	-5.690	-3	2	0.781	-	-	-	-
IGF-1-M	0.466	- 1	2	0.619	-	-	-	-
Gender	366	-56	788	0.082	-	-	-	-

PDGF-NB: Platelet derived growth factor-New born, PDGF-M: Platelet derived growth factor-Mother, IGF-BP3-NB: Insulin-like growth factor binding protein-3 New born, IGF-BP3 M:Insulin-like growth factor binding protein-3 Mother, FGF-2 NB: Fibroblast growth factor-2 New born, FGF-2 M: Fibroblast growth factor-2 Mother, GH-NB: Growth hormone-New born, GH M: Growth hormone- Mother, EGF-NB: Epidermal growth factor-New born, IGF-1 NB: Insulin-like growth factor-I New born, IGF-1 M: Insulin-like growth factor-I-Mother, infant's gender (male/female). Proposed model for the total sample: BW (g) = 5.041 × PDGF-M + 2.743 × FGF2-M + 7.673 × FGF2-NB-1264. GH should be considered as pituitary-GH.

## Discussion

An increased rate of foetal growth leading to macrosomia is the main abnormality in the infants born to GDM mothers [18]. In the present study, the GDM women were hyperinsulinemic and hyperglycemic, reflecting a decrease in insulin sensitivity in these individuals [19]. However, the macrosomic infants were only hyperinsulinemic. Indeed, it has been shown that during GDM, the mother's glucose, after its passage via the foeto-placental barrier, induces the release of insulin from foetal pancreas and, thereby, produces foetal hyperinsulinemia [19]. Hence, the increased levels of foetal insulin have been shown to stimulate mitogenic and anabolic mechanisms in the insulin sensitive foetal tissues, *i.e*., muscle, connective tissue and adipose tissue.

Recent studies have documented that, in addition to insulin, a variety of maternal and foetal insulin-like growth factors (IGF) may play an important role in the foetal growth [20]. The protocol of the present study did not allow us to discriminate into which compartment placentally produced hormones were secreted. However, we observed that serum IGF-I levels in macrosomic newborns and their GDM mothers were higher than those in controls. Our observations corroborate several reports which have demonstrated increased levels of IGF-I during pregnancy in both the maternal and foetal serum; hence, the upregulated IGF-I concentrations were correlated with the infant's birth weight [20-22]. In contrast, Hill et al. [23] have demonstrated no significant alterations in the levels of cord blood IGF-I of GDM mothers compared to the control. Interestingly, the expression of IGF-I mRNA is not significantly altered in the placenta of GDM mothers. In fact, Roth et al. [13] have demonstrated no differences in the expression of IGF-I mRNA in the placentas of GDM and control mothers. IGF-I cannot cross the placental barrier [24] and, hence, it is unlikely that the increase in the foetal size may be due to high maternal IGF-I levels. Though the origin of IGF-I in macrosomic babies is not well understood, high levels of foetal IGF-I may be implicated in the weight gain in the macrosomic babies [25].

The effect of delivery upon circulating hormone levels in general is unknown. In mother with GDM, IGF-I levels were significant correlated with IGF-I levels in macrosomic newborns (*r *= 0.52, *n *= 30, P = 0.017). Additionally, IGF-I might also influence the transport of glucose and amino acids across the placenta [25,26] and might again contribute to weight gain in macrosomic infants. Furthermore, the increased expression of IGF-IR mRNA in the placenta will also facilitate the mechanism of action of IGF-I in macrosomic babies. Our idea can be supported by the observations of Bhaumick et al. [27] who have reported a high placental number of IGF-IR during diabetic pregnancy. Hence, increased numbers of placental IGF-IR were supposed to be induced by the poor glycemic control as observed in the GDM women in our study [27].

Primarily, IGF-1 is regulated by the IGF-BP3, an IGF-I binding protein. The IGF-BP3 complexes with IGF-I and, therefore, acts as a reservoir for IGF-I in the blood circulation [28]. We have observed that IGF-BP3 levels are increased in the serum of GDM mothers and their macrosomic infants and IGF-I levels were significant correlated with IGF-BP3 in macrosomic newborns (*r *= 0.36, *n *= 30, P = 0.04). Our observations are in close agreement with the results of several investigators [29] who have demonstrated an increase in cord serum IGF-BP3 concentrations in GDM pregnancies, though Hill et al [23] have observed no significant modifications in the levels of IGF-BP3 of GDM mothers and their babies. In our study, the high levels of IGF-BP3 in GDM and their macrosomic infants are not contributed by the placenta as the IGF-BP3 mRNA expression is downregulated in GDM women. Hence, it is possible that high insulin and IGF-I concentrations both in GDM mothers and macrosomic babies might be responsible for high IGF-BP3 synthesis. Indeed, insulin has been shown to regulate IGF-BP3 levels in GDM pregnancy [30]. Moreover, insulin and IGF-I treatment *in vitro *also stimulates the secretion of IGF-BP3 [31].

Regarding GH, we would like to stress that placental GH constitutes the majority of circulating growth hormone in late pregnancy, and this hormone is not found in the fetal circulation. In our study, we observed that pituitary GH levels were significantly diminished in the GDM women and their macrosomic infants. Similarly, pituitary GH mRNA expression is also curtailed in the placenta of these women, though placental pituitary GHR mRNA is upregulated in these subjects. We would like to recall that pituitary GH levels are diminished gradually as a function of progress of a normal pregnancy [32]. It is noteworthy that placenta expresses another variant of GH (GHv) which bears a close homology with pituitary GH. Hence, we cannot elaborate the role of GHv. In our study, the PCR primers used for the GH could not distinguish between these two variants of GH. However, it is clear that they share 93% of amino acid sequences. Interestingly, in mother with GDM, pituitary GH levels were significant correlated with macrosomic birth weight babies (*r *= 0.36, *n *= 30, P = 0.04). How pituitary GH concentration in GDM and macrosomic babies are downregulated is not well understood. However, Lee et al. [33] have shown that insulin resistance, marked by high plasmatic insulin concentrations, is associated with low GH levels in diabetic subjects. It is possible that high IGF-I levels may be responsible for low GH concentrations in the GDM women and macrosomic babies. Our statement is supported by the observations of Lacroix et al. [34] who have demonstrated that placental exposure to high IGF-I levels could induce a down-regulation of pituitary GH production in the sheep. Besides, Misra et al. [35] have recently shown that low GH levels are involved in weight gain.

EGF has been shown to modulate foeto-placental growth regulation [34,36]. We observed that EGF concentrations were increased in GDM mothers and macrosomic babies though the placental EGF mRNA was not altered. Our results are in accordance with the report of Loukovaara et al. [37] who have shown that cord serum EGF concentrations are increased in GDM pregnancies. Since EGF-R mRNA expression is higher in the placenta of GDM women than that in control women, we can allude that high availability of these receptors in the presence of high EGF concentrations may be involved in the weight gain in the macrosomic babies. Hence, we would like to cite the results of Masuyama et al. [38] who have illustrated that EGF promotes amino acid transport in the rat placenta and, therefore, influences foetal growth. EGF has been reported not to cross the placental barrier [39]. How EGF concentrations are increased in GDM is not well-understood. However, it has been hypothesized that a rise in EGF levels seems to be a metabolic response of the foeto-placental unit to diabetes-related hyperglycemia [37]. In mother with GDM, glucose levels were significant correlated with EGF levels (*r *= -0.57, *n *= 30, P = 0.027) and with newborns EGF levels (*r *= - 0.51, *n *= 30, P = 0.031).

In our study, we observed higher FGF-2 concentrations in GDM mothers and their macrosomic infants than their respective controls. Furthermore, in the placenta of GDM women, we noticed a high expression of FGF-2 mRNA which may contribute to high FGF-2 synthesis. Our observations are substantiated by the report of Hill et al. [40] who have shown the placenta to be the site of FGF-2 synthesis. FGF-2, widely expressed by embryonic tissues [41], is associated to increased incidence of foetal macrosomia [42]. Whatsoever, the FGF-2R mRNA expression is downregulated in the placenta. Platelet-derived growth factor (PDGF) and its receptors (PDGFR) are important regulators for tissue interactions to cell migration, proliferation, survival and deposition of extracellular matrix during mammalian embryonic development [43]. Three ligands (PDGF-A, -B, and -C) bind to PDGFR-α with high affinity. To date, studies suggest that PDGF-B and PDGF-D mainly bind to PDGFR-β which is implicated in the organogenesis, whereas PDGF-A binds to PDGFR-α which is required for embryogenesis [43]. PDGF-C binds to both PDGFR-α and PDGFR-β and has limited role in the development of the foetus [43]. Since PDGF-B and PDGFR-β are also essential for development of normal structure and function of conduit vessels and capillaries, [44] we, in the present study, focused our study on these two parameters. Hence, we observed a significant rise in the mRNA expression of PDGF-B and PDGFR-β in the placenta of GDM mothers compared with controls. Plasma PDGF-B concentrations were also increased in GDM mothers and their macrosomic infants. The macrosomic birth weight were significant correlated with the plasma PDGF-B levels (*r *= -0.45, *n *= 30, P = 0.049). Such an increase in PDGF-B production in gestational myometrium could be associated with the uterine smooth muscle cell hyperplasia and hypertrophy, characteristics of the gestational uterus. Our results are in accordance with those obtained by Heiring et al. [45] who have observed a significantly stronger PDGFR mRNA in pregnant women with GDM compared with normal pregnant women. As far as the physiological inductor of PDGFR-β levels is concerned, we would like to mention the implication of insulin. The level of insulin in macrosomic newborns, were significant correlated with the plasma PDGF-B levels (*r *= 0.53, *n *= 30, P = 0.017). Indeed, insulin has been shown to enhance the mitogenic effects of PDGFR-β in cultured smooth muscle cells where it interferes with the cell signaling cascade, particularly with phosphatidylinositol-3-kinase of PDGFR-β [46,47]. High PDGF-B levels via PDGFR-β may also participate in placental angiogenesis in GDM women [48]. To properly interpret our results, we tried to present a linear regression model that summarizes all interactions between the different growth factors studied: BW (g) = 5,041 × PDGF-M 2743 + × FGF2-M 7673 + × FGF2-NB - 1264. Hence, the main factors that affect fetal weight are maternal PDGF, and maternal and fetal FGF2 (Table 3).

## Conclusion

A perusal of our observations suggests that human GDM and macrosomia are associated with down-regulation of GH and up-regulation of several growth factors, principally IGF-I, EGF, FGF-2 and PDGF-B. The mRNA expression of three growth factor receptors, *i.e*., IGF-IR, EGF-R and PDGFR-β, was upregulated in the placenta of GDM women. It seems that growth factors and their receptors influence materno-foeto-placental communication which might be implicated in the foetal weight gain in macrosomic babies during hyperinsulinemia, apparently seen in the GDM and their infants. However, further studies are required to study the interaction of growth factors with their receptors and whether downstream signaling cascade is altered in the placenta or target organs of GDM and macrosomia.

## Abbreviations

BMI: Body mass index; BW: Body weight; BL: Body length; CP: Cranial Parameter; GDM: Gestational Diabetes mellitus; EGF: Epidermal Growth factor; EGF-R: Epidermal growth factor receptor; FGF-2: Fibroblast growth factor-2; FGF-2R: Fibroblast growth factor-2 receptor; GDM: gestational diabetes mellitus; GH: Growth hormone; GHR: Growth hormone receptor; IGF-I: Insulin-like growth factor-I; IGF-IR: Insulin-like growth factor-I receptor; IGF-BP3: Insulin-like growth factor binding protein-3; Mac: macrosomic; PDGF-B: Platelet derived growth factor-β; PDGFR-β: Platelet derived growth factor receptor; HbA1c: Hemoglobin glycoside or clique; LSD: least significant difference; RQ: Relative quantity; CRP: C-Reactive Protein; HDL: high density lipoprotein; LDL: Low density lipoprotein; TG: Triglyceride; BW: Birth Weight; OGTT: oral glucose tolerance test.

## Competing interests

The authors declare that they have no competing interests.

## Authors' contributions

GO was in charge of the practical work and prepared major parts of the manuscript. AY and IM collected and analyzed data. AH contributed to the development of the protocol. DA-G, AG, FD and KM contributed to the collection of samples and the laboratory work. AM conducted biochemical analyses. HK participated in interpretation of the gynecology function. MZ carried out the immunoassays. AZ participated in the interpretation of the medical parameters. ZT and NAK planned and supervised the study and contributed to the revisions and the final drafts of the manuscripts.

All authors read and approved the final manuscript.

## Pre-publication history

The pre-publication history for this paper can be accessed here:

http://www.biomedcentral.com/1471-2393/10/7/prepub
